# Late Glacial summer paleohydrology across Central Europe

**DOI:** 10.1038/s41598-024-83189-7

**Published:** 2024-12-18

**Authors:** Maximilian Prochnow, Johannes Hepp, Paul Strobel, Roland Zech, Sudip Acharya, Sönke Szidat, Damien Rius, Laurent Millet, Bruno Glaser, Michael Zech

**Affiliations:** 1 Physical Geography, Institute of Geography, Friedrich Schiller University Jena, Jena, Germany; 2 Soil Biogeochemistry, Institute of Agronomy and Nutritional Sciences, Martin-Luther-University Halle-Wittenberg, Halle, Germany; 3 Physical Geography, Institute of Geography, Technical University Dresden, Dresden, Germany; 4 Department of Chemistry, Biochemistry and Pharmaceutical Sciences, Oeschger Centre for Climate Change Research, University of Bern, Bern, Switzerland; 5 Laboratoire Chrono-Environnement, UFR des Sciences et Techniques, CNRS UMR 6249, Université de Bourgogne-Franche-Comté, Besançon, France; 6 Department of Geology, University at Buffalo, Buffalo, NY USA

**Keywords:** Deuterium excess, Evapotranspiration, Seasonality, Stable isotopes, Younger Dryas, Preboreal, Biogeochemistry, Palaeoclimate

## Abstract

It is generally accepted that a weakening of the North Atlantic thermohaline circulation caused the Younger Dryas cooling. Although the role of seasonality was emphasized previously, this aspect is rarely considered yet, and it remains elusive how this impacted hydroclimate during winters and summers across Central Europe. Here, we coupled biomarker-based δ^18^O and δ^2^H from Bergsee in southern Germany to reconstruct deuterium excess as a proxy for evaporation history from the Bølling-Allerød to the Preboreal. We compared this dataset with other biomarker isotope records in Central Europe. They are all lacking a strong isotopic depletion during the Younger Dryas, which is best explained by the summer sensitivity of the biomarker proxies: As Younger Dryas summers were relatively warm, there is an absence of the strong winter cooling signals recorded in annual water isotope records like Greenland or Lake Steißlingen. Lake evaporation at Bergsee together with other paleohydrological reconstructions draw a coherent picture of the Late Glacial hydroclimate, with strong evidence for warm and dry Younger Dryas summers. Rather than a southward shift of the Westerlies during winter, we suggest that a recently proposed feedback mechanism between North Atlantic sea ice extend, strong winter cooling and summer atmospheric blocking serves as a suitable explanation for summer dryness. Additional confidence to the robustness of these biomarker records is provided by the overall agreement of paleohydrological fluctuations during the Preboreal.

**Supplementary Information:**

The online version contains supplementary material available at 10.1038/s41598-024-83189-7.

## Introduction

The Late Glacial (18 ka – 11.7 ka BP) is one of the most extensively studied periods in paleoclimatology. Stable isotope analyses from Greenland ice cores provide a robust understanding of the timing and magnitude of abrupt temperature changes during the Late Glacial^1–3^. It marks a rapid warming during the Bølling-Allerød interstadial (14.7–12.8 ka BP), interrupted by a sudden shift to cooler conditions called the Younger Dryas (12.8–11.7 ka BP). It is generally accepted that the Younger Dryas cooling was associated with meltwater discharge from northern hemispheric ice sheets and a subsequent weakening of the thermohaline circulation in the North Atlantic^4–6^. Several studies have emphasized the importance of seasonality during the Late Glacial, i.e. particularly cold and harsh winters^4,5,7^, yet it remains unclear how this impacted hydroclimatic conditions during winters and summers, respectively, across Central Europe.

Most paleohydrological records are based on lake level reconstructions^8,9^ or stable isotopes from ostracods^10^, carbonates^11,12^ or lipid biomarkers^13–15^ preserved in lake sediments. Lipid biomarkers are resistant against degradation across a broad variability in climate conditions^16^ and in sedimentary archives, they remain preserved over long geological time scales^17^. The most commonly used lipid biomarker proxy is compound-specific δ^2^H on *n*-alkanes. In lakes, long chain *n*-alkanes (i.e., *n*-C_27_ to *n*-C_31_) originate from leaf waxes of terrestrial plants, and their compound-specific δ^2^H signal records the isotope composition of precipitation during growing season^18^. *n*-C_31_ mainly originates from grasses, and its δ^2^H signature refers to the isotope composition of precipitation without a strong modulation by transpirative enrichment due to plant physiology^19,20^. *n*-C_27_ and *n*-C_29_ are mainly produced by bushes and trees. Their compound-specific δ^2^H signature is also driven by the isotope composition of precipitation, but it is further influenced by transpirative leaf water enrichment. Mid-chain *n*-alkanes (e.g., *n*-C_21_, *n*-C_23_) are attributed to aquatic organisms such as submerged aquatic plants, reflecting the δ^2^H signature of lake water^18,21^. Depending on the setting, lake water evaporation can alter the δ^2^H signature of *n*-alkanes. Comparing the isotope signatures of *n*-alkanes from different sources can enable the reconstruction of past evapo(transpi)ration, which provides very valuable information about drought and moisture availability^22–25^. Meerfelder Maar in Germany^15^ and Hässeldala Port in South Sweden^14^ are two pioneer lake sediment records utilizing this “dual biomarker approach”^23^ to reconstruct leaf water transpirative enrichment during the Younger Dryas.

Over the last years, the so-called “coupled isotope approach” was developed making additional use of compound-specific δ^18^O analyses on hemicellulose sugars^13,26,27^. Like *n*-alkanes, they derive either from aquatic or terrestrial sources^28^. The combination of both biomarker stable isotopes (δ^2^H of *n*-alkanes and δ^18^O of sugars) allows to reconstruct deuterium excess, which is a direct proxy for evapo(transpi)rative enrichment^27^. Coupled isotope approaches have a major added value compared to single isotope approaches, as they allow to disentangle between changes in the isotope signature of precipitation and other isotope effects such as evapo(transpi)rative enrichment. In Europe, this approach was successfully applied at Bichlersee in Bavaria^29^ and Gemündener Maar close to Meerfelder Maar^13^.

It must be emphasized that the successful application of dual and coupled biomarker approaches depends on a clear source identification of aquatic and terrestrial biomarkers and, depending on the archive and setting, this is not always unambiguous^30^. So far, however, such studies use partly different approaches, are only selectively compared with each other, and hydroclimatic conditions during the Late Glacial-Early Holocene transition across Central Europe are still controversially discussed^13,15,29,31,32^. Moreover, the aspect of seasonality is only rarely considered yet. The picture of the Late Glacial paleohydrology across Central Europe thus remains elusive.

In this study, we revisited an existing biomarker stable-isotope record from Bergsee, Black Forest, South Germany, described previously by Hepp^30^. This record covers the Late Glacial–Early Holocene (~ 15 to 11 ka cal. BP) and consists of a high-resolution compound-specific δ^2^H_*n*−alkane_ and δ^18^O_sugar_ dataset. Yet, the application of the dual and the coupled isotope approach was not possible due to a questionable source attribution of *n*-alkanes and sugars (aquatic versus terrestrial) and unclear impacts of seasonality and various isotope effects. In this regard, one objective of this study is to re-evaluate δ^2^H and δ^18^O as hydrological proxies and to couple both isotopes, allowing to calculate deuterium excess as a proxy for evapo(transpi)rative enrichment. By comparing our newly established record to existing stable-isotope records from northern Europe and the Alps, we evaluate the spatio-temporal consistency of biomarker-based paleohydrological patterns across Central Europe.

## Study site and sediment record

**Fig. 1:**
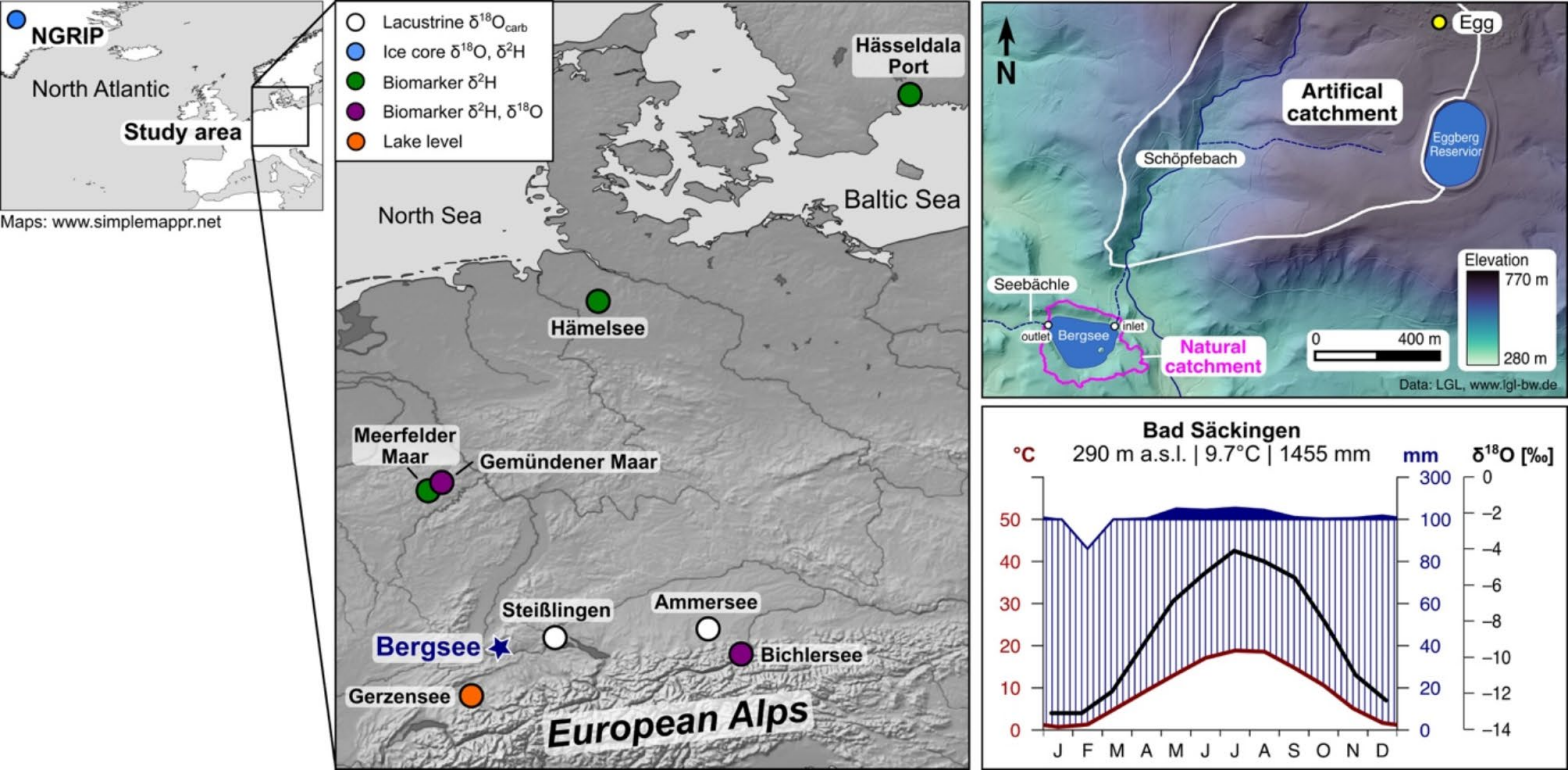
Geographic overview of Bergsee. The maps on the left indicate locations and types of paleohydrological records across Central Europe mentioned in the text. The right map shows Bergsee and its hydrological setting (Data: LGL, www.lgl-bw.de) and the climate diagram of Bad Säckingen illustrates climate conditions (Data: www.climate-data.org) and provides the monthly isotopic composition of δ^18^O in precipitation for the area^35,36^. The maps were made with SimpleMappr (www.simplemappr.net) and QGIS 3.4 (www.qgis.org). The figure was created with Inkscape 1.3.2 (www.inkscape.org).

Bergsee (Fig. 1) is located in a small depression in the southern Black Forest, Southern Germany (47°34’20’’N 7°56’11’’E, 380 m a.s.l), surrounded by dense forest consisting of *Abies* sp. and *Fagus* sp.^33^. It covers a surface area of ~ 8.25 ha and has a maximum water depth of ~ 13 m. It is mainly fed by precipitation and little groundwater^34^ as it has no surficial inflow^33^. Bergsee has only a small overflow forming the creek Seebächle. The natural catchment is limited to the slopes of its depression and covers ~ 0.16 km^2^. However, an artificial channel was established in 1802 CE extending its modern catchment to ~ 10 km^2^ (Fig. 1)^33^. The geology of the catchment consists of paleozoic gneisses and granites^33^, on which cambisols have developed. The modern climate conditions of the site are illustrated with a climate diagram from Bad Säckingen, a town 2 km south of Bergsee (Fig. 1). The mean annual air temperature is 9.7 °C, and the mean annual precipitation is 1455 mm. ^18^O in precipitation is enriched during the summer months and more depleted during winter, ranging from − 4‰ in July to − 12‰ in December^35,36^.

**Fig. 2:**
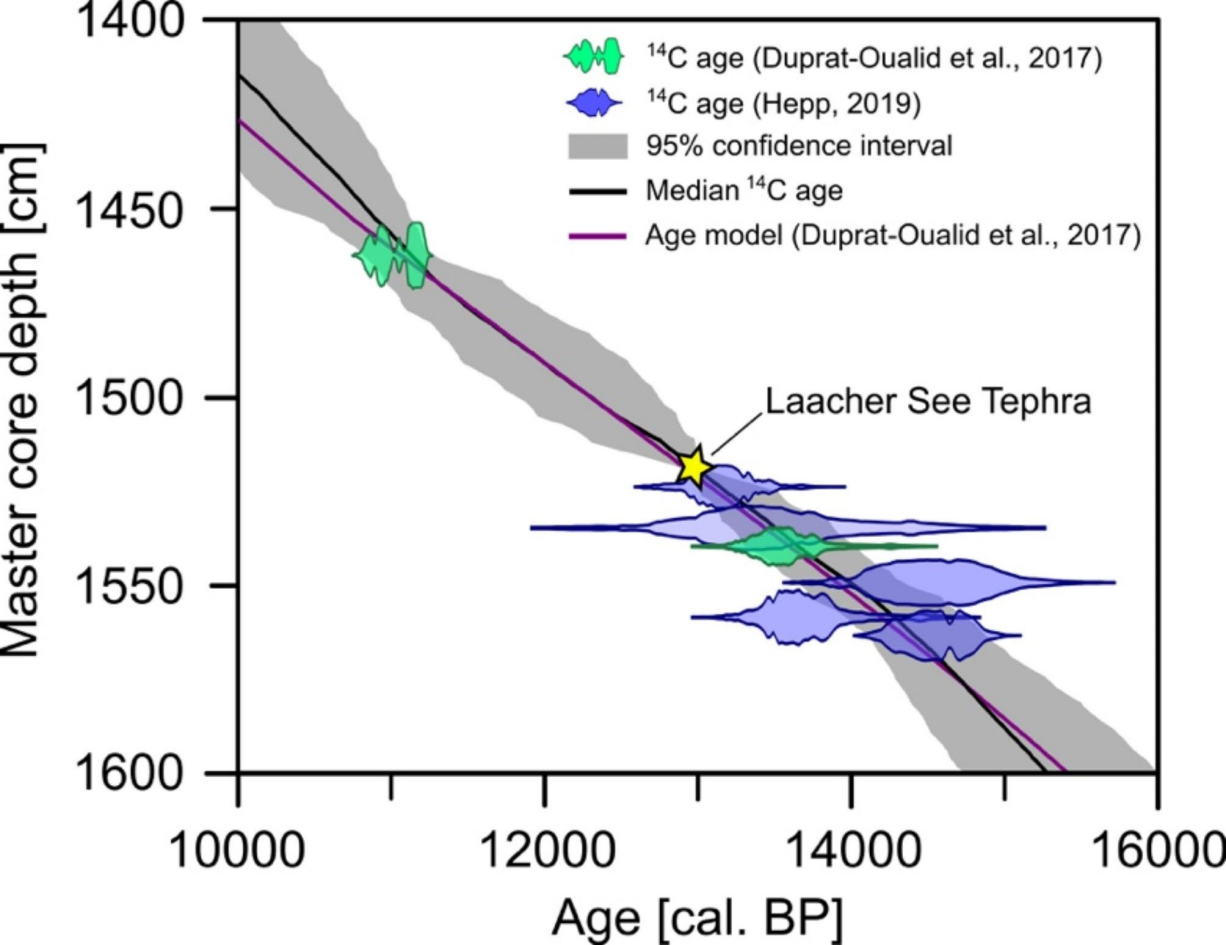
Revised age-depth model for the Late Glacial section of the Bergsee sediment record. The figure was created with Inkscape 1.3.2 (www.inkscape.org).

This study is based on a lake sediment core retrieved in 2013. A first chronology and palynological results were published by Duprat-Oualid, et al.^37^. The chronology of the Late Glacial part (from 1450 to 1605 cm) was then refined with five additional macrofossil ^14^C ages measured at the LARA AMS laboratory at the University of Bern^30,38^. The age-depth model was further re-calculated by applying the IntCal20 calibration curve^39^ in rBacon 2.5.8^40^. This revised age-depth model shows slight differences compared to the original chronology (~ 70 years from 1450 to 1470 cm, ~ 40 years from 1470 to 1570 cm, ~ 100 years below 1580 cm; Fig. 2).

The Bergsee *n*-alkane and hemicellulose biomarker and stable isotope dataset re-evaluated in this study is based on sediment material continuously taken from 1450 to 1605 cm master core depth in 1 cm slices^30^. For information about the biomarker analyses the interested reader is referred to Hepp^30^. To ensure quality control, we report only results with analytical uncertainty (standard error) better than 6‰ for δ^2^H and 0.7‰ for δ^18^O based on at least triplicate analyses. The biomarker datasets have a mean temporal resolution of ~ 41 years for δ^18^O_sugar_ and ~ 32 years for δ^2^H_*n*−alkanes_.

## Results and discussion

### Compound-specific isotope composition of leaf wax and sugar biomarkers and their seasonal implications

**Fig. 3:**
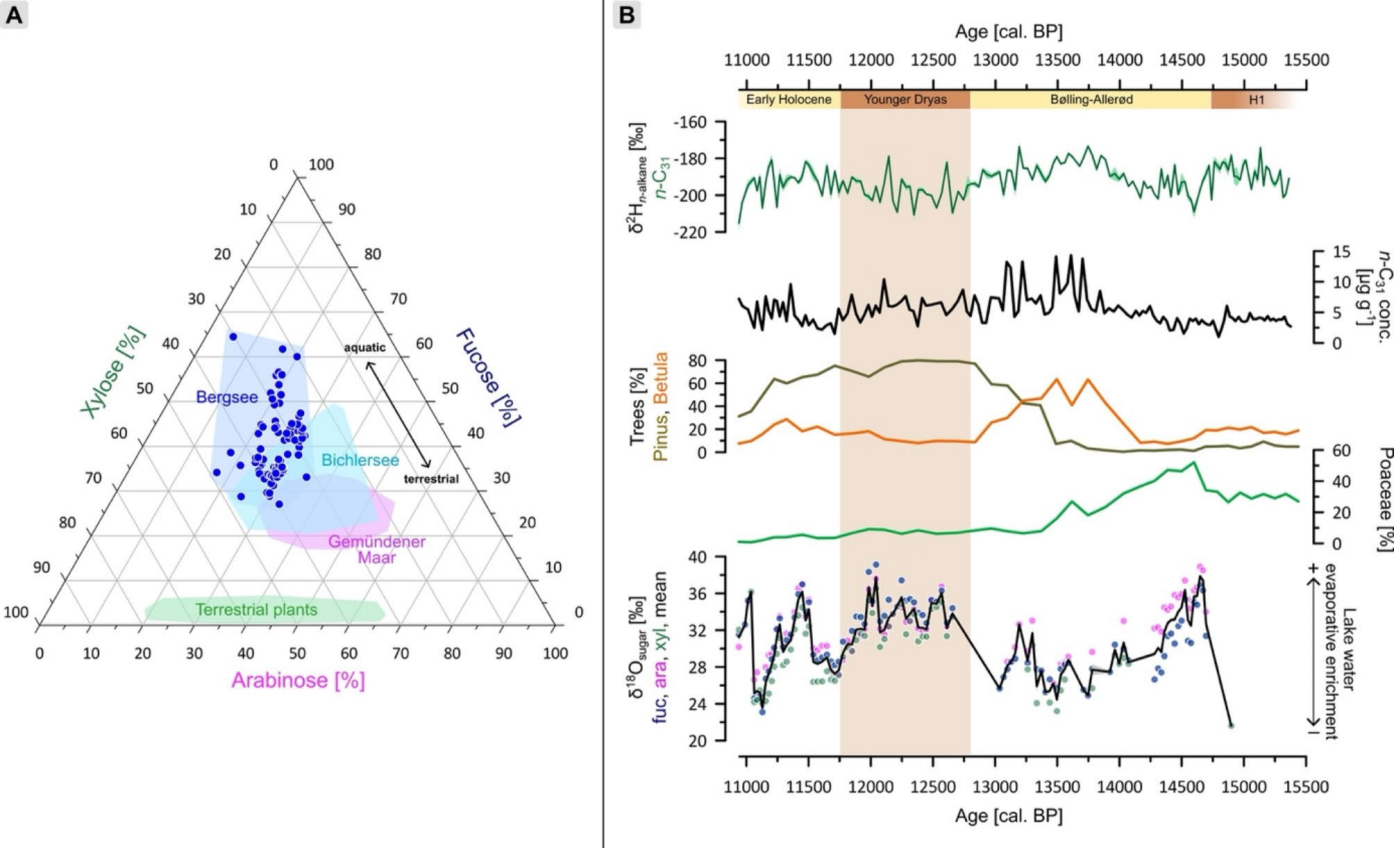
Source evaluation of *n*-alkane and sugar biomarker. **(A)** Ternary diagram illustrating abundances of arabinose, fucose and xylose in the Bergsee sediments^30^ compared with data from Gemündener Maar^13^, Bichlersee^29^ and terrestrial plants^28^. **(B)** Results of compound-specific δ^2^H and δ^18^O analyses on *n*-alkanes and hemicellulose sugars, respectively. Colored ribbons resemble analytical uncertainty expressed as standard error. The concentration of *n*-C_31_ and pollen of *Betula*, *Pinus* and *Poaceae* are used to indicate the vegetation composition at Bergsee^37^. The figure was created with Inkscape 1.3.2 (www.inkscape.org).

Here, we focus on δ^2^H of *n*-C_31_ only, because the source attribution of the other *n*-alkanes (in particular *n*-C_23_ and *n*-C_25_) is challenging and the dual isotope approach using aquatic versus terrestrial δ^2^H isotope signals is not possible^30^. δ^2^H of *n*-C_31_ ranges from − 215 to − 175‰, with less ^2^H-depleted values during the Bølling-Allerød and Early Holocene and more depleted values during the Younger Dryas (Fig. 3B). The interpretation of δ^2^H depends on the *n*-alkane source. Long chain homologues such as *n*-C_31_ are generally attributed to terrestrial vegetation^41^. Grasses are known to produce high amounts of *n*-C_31_^42–44^. Despite the strong forestation by the typical Late Glacial pioneer vegetation *Betula* and *Pinus* during the Bølling-Allerød, such forests have a grassy understory. Even during periods of stronger tree cover, it can be assumed that *n*-C_31_ is still dominantly derived from grasses. This is supported by high abundances of *Poaceae* pollen (~ 10 to 50%) and high *n*-C_31_ contents (> 5 µg g^− 1^) for the Bølling-Allerød and during the Younger Dryas in the Bergsee sediments (Fig. 3B). There is also no change of δ^2^H *n*-C_31_ coinciding with higher abundances of trees, which would indicate a contribution of trees (which show transpirative enrichment) to *n*-C_31_. We thus argue that δ^2^H of *n*-C_31_ is a robust endmember for grasses. Our study site receives rainfall throughout the year, and growing season precipitation accounts for ~ 64% of annual precipitation. Grasses are photosynthetically active only during the growing season and leaf wax synthesis is therefore mainly influenced by δ^2^H of summer precipitation. Enriched values of δ^2^H are also partly attributed to leaf water transpiration. However, we argue that this effect is only of minor importance as grasses are not strongly affected by leaf water transpiration due to their specific plant physiology^19^. Thus, δ^2^H of *n*-C_31_ likely refers to the isotope composition of growing season precipitation (ca. June to end of August during the Younger Dryas^5^).

Like δ^2^H of *n*-alkanes, the interpretation of δ^18^O of hemicellulose-derived sugars also depends on the compound source. The ternary diagram in Fig. 3A shows that Bergsee sediments yield high abundances of fucose (~ 40%), but lower amounts of arabinose (~ 25%) and xylose (~ 35%). Moreover, the fucose / (arabinose + xylose) ratio yields values ranging from 0.4 to 4.5, indicating a primarily aquatic origin of all three sugars^28,30^. This is a typical finding for lacustrine sediments as reflected by sugar pattern data including sediments and modern plant data from Gemündener Maar in northern Germany and Bichlersee, Bavarian Alps^13,28,29^. In contrast to terrestrial plants, which contain mainly arabinose and xylose, aquatic organisms like diatoms and zooplankton produce large amounts of fucose^45^. δ^18^O of fucose, arabinose and xylose show very similar patterns, ranging from 23‰ to 40‰ (Fig. 3B). The comparable pattern provides additional evidence that all three sugars likely primarily originate from identical and thus aquatic sources. Aquatic organisms primarily use lake water for biosynthesis of hemicellulose sugars^46^, so we interpret the mean of δ^18^O of all three sugars (δ^18^O sugar) as a proxy for the isotope composition of lake water. As the highest production of aquatic sugars is coupled to lake productivity during the growing season, it can be assumed that δ^18^O sugar is sensitive for the isotope composition of lake water during summer. Moreover, it must be considered that before the 19th century Bergsee was less than 10 m deep, had a very small catchment (0.1 km^2^) and due to the geology only minor groundwater inflow^34^ and no outflow; so the strong influence of evaporative enrichment on δ^18^O sugar is likely in this setting^30^. The δ^18^O record is further discussed in the context of the deuterium excess in “Deuterium excess as a proxy for evaporative enrichment”.

The importance of seasonality regarding the interpretation of traditional (e.g., ice cores, carbonates) and biomarker stable-isotope records was emphasized in several studies^4,29,47–51^. It affects many aspects of climate, for example, temperature, evapo(transpi)ration or even atmospheric circulation, and thus influences how different proxies respond to such seasonal differences. This implies specific limitations regarding the comparability between certain types of stable-isotope records. However, especially for biomarker records across Central Europe covering the Late Glacial, this aspect is rarely considered although this period is characterized by strong seasonality^4,6,7^. In the following, we attempt to combine the biomarker data from Bergsee and other isotope records from Central Europe by considering the perspective of seasonality during the Late Glacial.

**Fig. 4:**
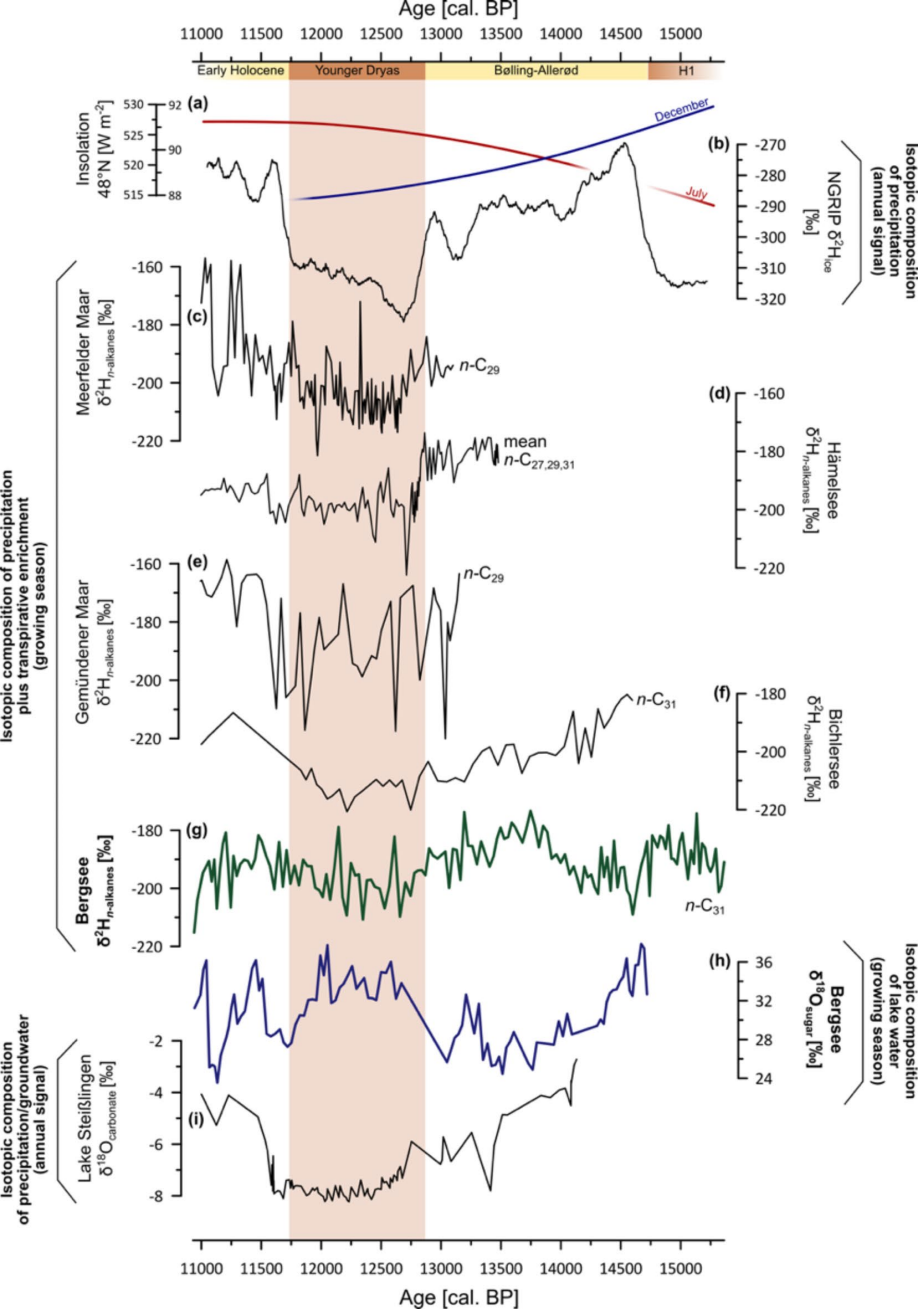
Compilation of δ^18^O and δ^2^H records. (**a**) December and July insolation^59^ reflect seasonality during the Late Glacial. The annual precipitation signal is reflected by stable-isotope records from (**b**) Greenland ice cores^53^ and (**i**) Lake Steißlingen^11,12^. Note that δ^18^O from Ammersee^10^ as mentioned in the text looks similar to Greenland and Lake Steißlingen. In contrast, the Central Europe leaf wax biomarker δ^2^H records from (**c**) Meerfelder Maar^15^, (**d**) Hämelsee^52^, (**e**) Gemündener Maar^13^, (**f**) Bichlersee^29^ and (**g**) Bergsee reflect a growing season signal. The same holds true for our (**h**) δ^18^O sugar record from Bergsee reflecting lake water during summer. The figure was created with Inkscape 1.3.2 (www.inkscape.org).

The leaf wax biomarker δ^2^H records from Central Europe, i.e., Meerfelder Maar^15^, Gemündener Maar^13^, Hämelsee^52^, Bichlersee^29^ and Bergsee show a short-term variability during the Late Glacial (Fig. 4c-g). However, they share similar long-term trends, i.e., more ^2^H-enrichment during the Bølling-Allerød and the Early Holocene compared to the Younger Dryas. At Gemündener Maar, Meerfelder Maar and to some degree also at Hämelsee, the effect of transpirative leaf water ^2^H-enrichment needs to be considered, because leaf waxes (*n*-C_29_, *n*-C_27_) are likely derived from trees there^13,15^, whereas δ^2^H of *n*-C_31_ from Bergsee and Bichlersee reflect grasses without strong transpirative enrichment^20,29^. Despite such conceptual differences, we suppose that the similar long-term trends in all of those records are best explained by changes in the isotope composition of precipitation.

δ^2^H as well as δ^18^O from the NGRIP-record from Greenland (Fig. 4b), is traditionally interpreted to reflect the isotope signature of precipitation and is widely used as a temperature proxy in paleostudies^3,4,53,54^. In comparison with the Central European leaf wax δ^2^H records from Bergsee, Meerfelder Maar, Gemündener Maar, Hämelsee and Bichlersee, Greenland ice cores show a much stronger stadial interstadial pattern between the Bølling-Allerød and the Younger Dryas. All of these records show similar trends, but a sharp depletion during the Younger Dryas seems absent in the biomarker leaf wax records as previously discussed by Prochnow, et al.^29^. We argue that this is related to the different seasonal sensitivity^55^ of those isotope records: Greenland ice cores are high latitude polar records with a strong temperature effect on δ^2^H and δ^18^O^56^. They thus represent an annual signal including the strong arctic winter cooling^4^. In contrast, the Central European leaf wax δ^2^H records rather reflect the isotope composition of precipitation during growing season, i.e. they are proxies for summer water cycle^18,29,48^. Strong seasonal differences become most obvious by comparing winter and summer insolation (Fig. 4a). During the Late Glacial, northern hemispheric summer insolation is increasing, whereas winter insolation is decreasing due to precession forcing^57^. This pattern suggests relatively warm Younger Dryas summers. In fact, new climate simulations suggest that the Younger Dryas summers were as warm as during the Bølling-Allerød interstadial^5^, as discussed in more detail regarding our deuterium excess reconstruction in  “Deuterium excess as a proxy for evaporative enrichment”. This is likely the reason why our compilation of Central European leaf wax isotope records lack a strong temperature-related depletion of the cold season as shown by NGRIP^29^. However, the striking short- and long-term δ^2^H excursions in some of those biomarker records need to be addressed. While transpiration due to different biomarker sources (trees versus grasses) might play one role, it should be considered that all of those study sites have different hydrological settings, i.e., a different importance of groundwater inflow and throughflow, and more or less winter snow cover due to different catchment sizes. The latter is particular important for plants, because snow reflects the depleted isotope composition of winter precipitation and can influence the isotopic signature of soil water. This might explain why some of those leaf wax records still show a Younger Dryas depletion signal, albeit only a weak one, and a stronger scatter because snow cover remaining at the beginning of the short but warm growing seasons during the Younger Dryas would add a winter bias to the summer-sensitive leaf wax signals.

Comparing ice core and leaf wax stable-isotope records provides just one example regarding seasonality. It is noteworthy that it is also relevant among other sets of proxies: For instance, Mateo-Beneito, et al.^58^ discussed seasonal differences between specific annual GDGT and summer-sensitive chironomid temperatures during the Younger Dryas in lake sediments from the Bohemian Forest, whereas Daniels, et al.^48^ found differences in spring versus summer temperatures based on alkenones and leaf wax δ^2^H during the Late Glacial in Alaska.

The effect of seasonality becomes even more clear when comparing the Bergsee δ^18^O sugar data with other lacustrine δ^18^O records from the region. δ^18^O sugar of Bergsee can be compared with a regional carbonate δ^18^O record from Lake Steißlingen^11,12^ (Fig. 4i) and the benthic δ^18^O ostracod record from Ammersee^10^. These two lakes show more negative δ^18^O values during the Younger Dryas as known from Greenland, which is the opposite trend compared to our Bergsee δ^18^O sugar record (Fig. 4h-i). This discrepancy can be related to the different hydrology of those lakes and the resulting seasonal sensitivity of the δ^18^O records. Lake Steißlingen is mainly fed by annual groundwater inflow (integrating year-round rainfall) with a minor influence of evaporation^30,60^. Ammersee is a large lake with throughflow and a hypolimnion where lake water is reflecting the annual isotopic variations without a strong evaporation bias^51,61^. Accordingly, the δ^18^O signature derived from carbonates from Lake Steißlingen and benthic ostracods living in the hypolimnion at Ammersee reflect the precipitation signal integrated over the whole year. We assume that the isotopic signal of those lakes may not be a simple amount-weighted mean of precipitation, but slightly biased toward cold season because evapo(transpi)ration reduces the volumetric contribution of summer precipitation to both runoff and groundwater. This might be one reason why groundwater-based records of δ^18^O may disproportionately reflect winter over summer variations in meteoric δ^18^O. Therefore, δ^18^O from Lake Steißlingen and Ammersee can be considered as a close to annual but slightly winter-biased signal, while our δ^18^O sugar record from Bergsee is summer sensitive and additionally influenced by evaporative enrichment^29^.

### Deuterium excess as a proxy for evaporative enrichment

**Fig. 5:**
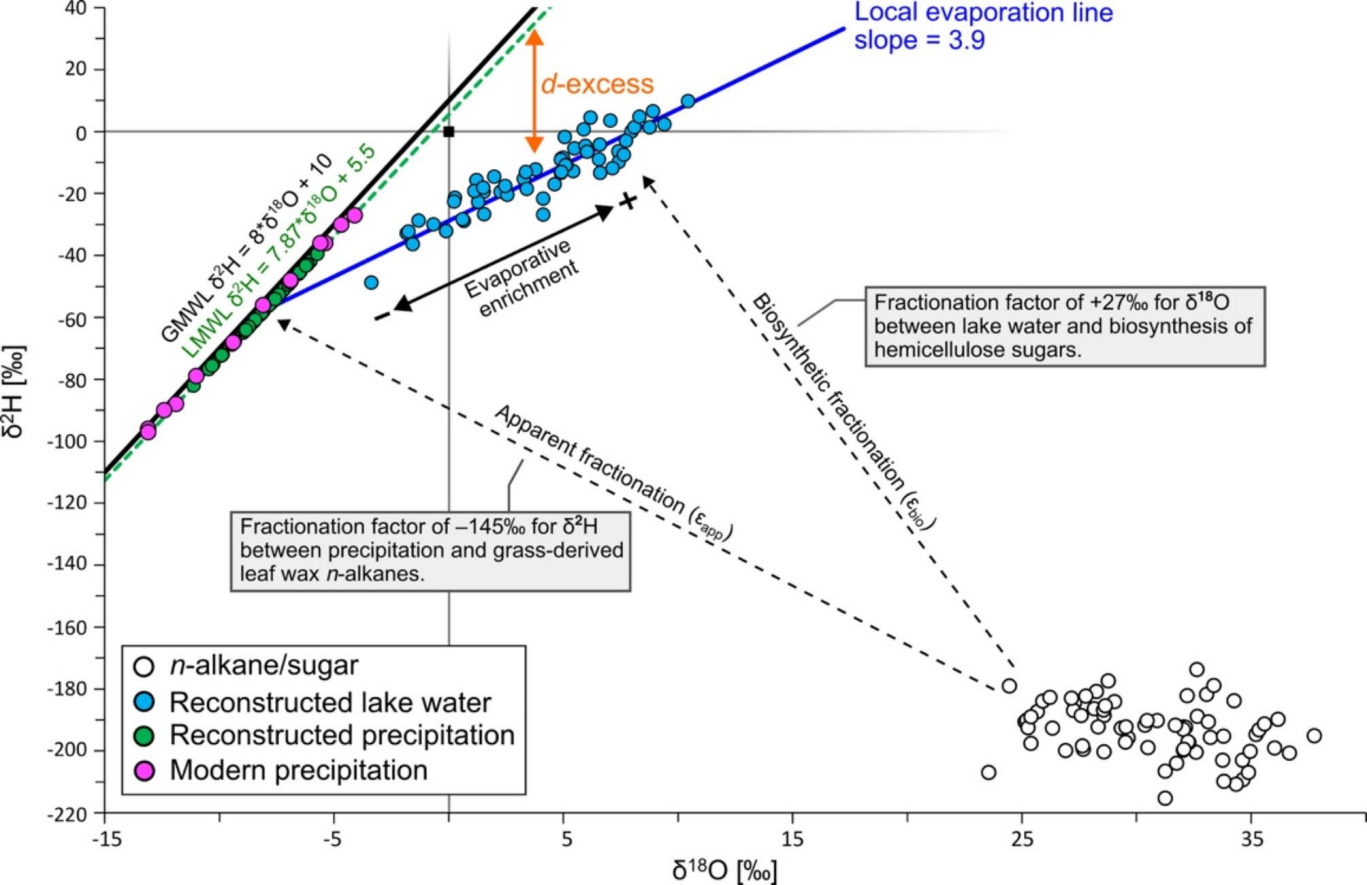
δ^18^O–δ^2^H cross plot showing the concept of the coupled isotope approach^29,64^. Datapoints resemble isotope values of biomarkers and the calculated isotopic composition of lake water and precipitation. Modern precipitation data is from Bowen and Revenaugh^35^ and Bowen, et al.^36^. The figure was created with Inkscape 1.3.2 (www.inkscape.org).

The relationship between δ^2^H and δ^18^O in precipitation can be described by the Global Meteoric Waterline (GMWL), or on a regional scale, a Local Meteoric Waterline (LMWL). For Bergsee, the LMWL of Weil am Rhein with δ^2^H=7.87 × δ^18^O+5.5^62^, 20 km west from our site, can be used. Rainfall that is “trapped” in lakes therefore plots close to this meteoric waterline. In our case, δ^2^H of precipitation can be calculated based on δ^2^H of *n*-C_31_ by applying the apparent fractionation (ε_app_) between source water and leaf wax *n*-alkanes of − 145 ± 12‰ (± 6‰ standard error) (Fig. 5), which was reported for grass sites along a transect across Central Europe^20^:

1 $$\:{{\updelta}}^{\text{2}}{\text{H}}_{\text{p\:}}{=}\left[\frac{\left(\frac{{{\updelta}}^{\text{2}}{\text{H}}_{\text{n-C31}}}{\text{1000\:+\:1}}\right)}{\left(\frac{{{\upepsilon}}_{\text{app}}}{\text{1000\:+\:1}}\right)}{\:-\:1}\right]{\times\:1000}$$

Using a fixed ε_app_ is a potential limitation of this approach, as it might be variable depending on specific climate settings. However, the value of − 145‰ used here should be a good approximation as it is within in the range of the average ε_app_ for grasses reported in a global dataset covering variable climate conditions (~–149‰)^18^ and a compilation from semi-arid Mongolia (~–142‰)^21,63^. We acknowledge that the reconstructed δ^2^H of precipitation (mean − 56‰, ranging from − 82 to − 40‰) is in good agreement with the actual modern growing season precipitation (April to September, mean − 41‰, ranging from − 68‰ to − 27‰; see Fig. 5). By combining the δ^2^H of precipitation and the LMWL, δ^18^O of precipitation can be estimated. Due to evaporation, lake water gets isotopically enriched in ^18^O and ^2^H along a so-called Local Evaporation Line (LEL). The LEL can be described with δ^2^H_lake_=*m* × δ^18^O_lake_+*n*, where *m* is the slope and *n* the intercept with the δ^2^H axis. Therefore, lake water shows an offset to the meteoric water line, while the ^2^H distance between the evaporatively enriched lake water and the meteoric water line is defined as deuterium excess – in this case a proxy for evaporative enrichment (Fig. 5).

Lake water δ^18^O can be calculated based on δ^18^O sugar by applying the biosynthetic fractionation (ε_bio_, Fig. 5) factor of ~ 27‰^65–67^:

2 $$\:{{\updelta}}^{\text{18}}{\text{O}}_{\text{lake\:}}{=}\left[\frac{\left(\frac{{{\updelta}}^{\text{18}}{\text{O}}_{\text{fucose}}}{\text{1000\:+\:1}}\right)}{\left(\frac{{{\upepsilon}}_{\text{bio}}}{\text{1000\:+\:1}}\right)}{\:-\:1}\right]{\times\:1000}$$

In the literature, ε_bio_ values range from 27 to 30‰ (*n* = 4)^65–68^, resulting in an uncertainty of ± 0.75‰ (standard error), while species-specific effects have no notable influence on ε_bio_^68^. Note that the corresponding δ^18^O lake water values (~ 4‰) are about ~ 10‰ more positive than δ^18^O of modern growing season precipitation (April to September, mean − 6‰, ranging from − 9‰ to − 4‰), suggesting additional evaporative enrichment.

**Fig. 6:**
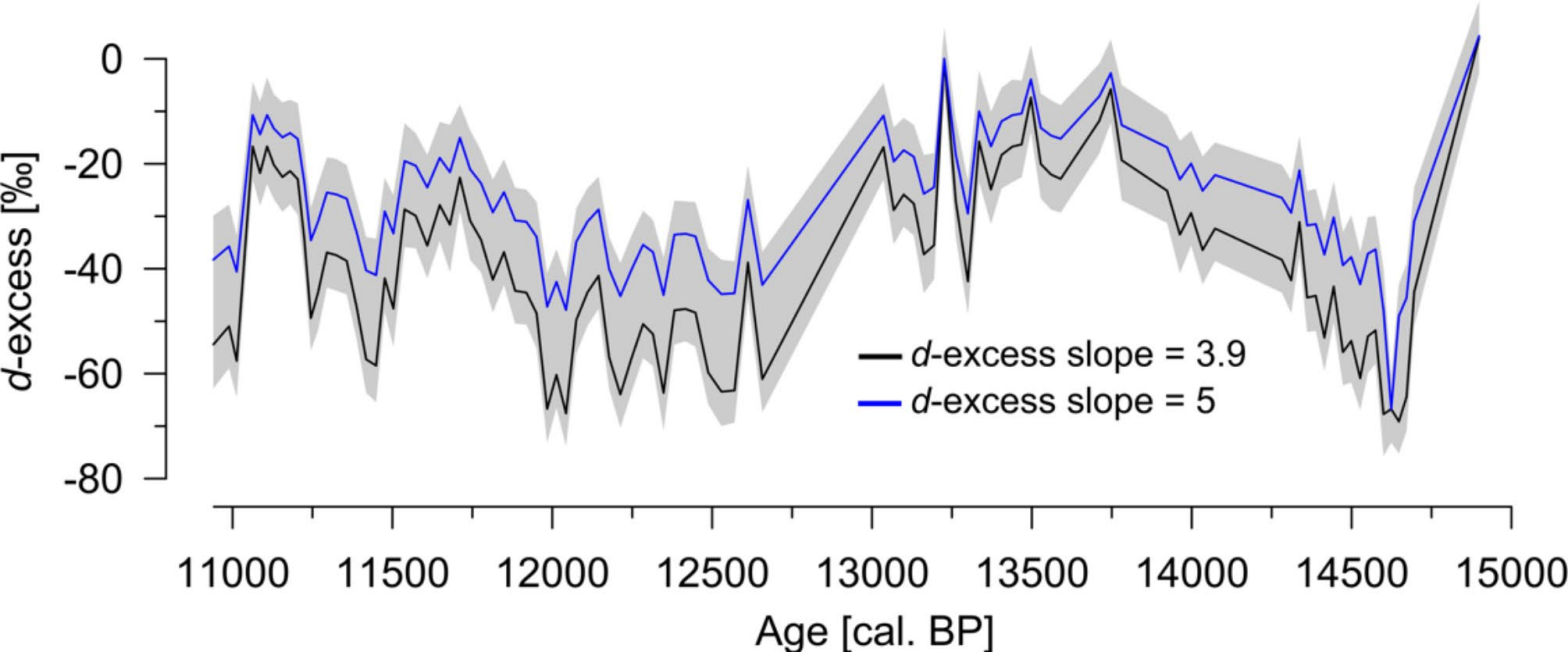
Deuterium excess reconstructions for Bergsee using different settings for the slope calculation. Grey ribbon indicates the uncertainty (standard error) of all calculations. The figure was created with Inkscape 1.3.2 (www.inkscape.org).

As the slope *m* of the LEL depends on temperature^69^, we used a mean growing season air temperature of 14.8 °C for Bergsee (360 m a.s.l), which is based on lapse-rate corrected temperature data from Bad Säckingen (280 m a.s.l.) by assuming a constant temperature gradient of − 0.6 K per 100 m. This yields a slope of 3.9, which is only slightly lower than typical slopes between 4 and 5 reported in the literature^61,70–72^. This difference might be related to the fact that those studies are based on modern climate conditions and integrate over different regions worldwide with changing relative humidity, which has an impact on the slope of the LEL^72,73^. While summer temperatures changed by a magnitude of ± 1 K during the Bølling-Allerød – Younger Dryas transition^5^, this has only a small effect on our deuterium excess reconstruction because LEL slopes are rather insensitive to temperature changes^29^. However, using the calculated and reported constant slopes from the literature has only a small impact on the amplitude of the deuterium excess reconstruction, while its overall trends remain similar (Fig. 6).

As the local evaporation line intersects the local meteoric waterline, the LEL’s intercept *n* can be calculated with Eq. (3) by using δ^2^H and δ^18^O of precipitation from Eqs. (1) and (2):

3 $$\:\text{n\:}{=\:}{\updelta}^{\text{2}}{\text{H}}_{\text{p}}\:{-(} \text{m}{\:\times\:}{\updelta}^{\text{18}}{\text{O}}_{\text{p}}{)}$$

With δ^18^O of lake water, the slope *m* and the intercept *n*, δ^2^H of lake water is calculated (Eq. 4), and by entering the δ^18^O and δ^2^H values of lake water in the meteoric water line, deuterium excess can be inferred (Eq. 5):

4 $$\:{\updelta}^{\text{2}}{\text{H}}_{\text{lake}}{=\:}\text{m}{\:\times\:}{\updelta}^{\text{18}}{\text{O}}_{\text{lake}}{+\:}\text{n}$$

5 $$\:\text{d}\text{-excess}\text{\:}{\:=\:}{\updelta}^{\text{2}}{\text{H}}_{\text{lake}}\:\--\text{(}\text{7.87}{\:\times\:}{\updelta}^{\text{18}}{\text{O}}_{\text{lake}}\text{)}$$

The uncertainty of deuterium excess as standard error can be calculated using standard errors (se) of δ^2^H_*n*−alkanes_, δ^18^O_sugar_ weighted by the LMWL slope, ε_bio_ and ε_app_^64^:

6 $${\text{s}}{{\text{e}}_{{\text{d - excess}}}}{{ = }}\sqrt {{\text{s}}{{\text{e}}_{{\text{2H}}}}^{\text{2}}{{ + }}\left( {{{7}}{{.87 \times }}{{\text{se}}_{{\text{18O}}}}^{\text{2}}} \right){{ + }}{{\text{se}}_{{{\upepsilon {\rm bio}}}}}^{\text{2}}{{ + }}{{\text{se}}_{{{\upepsilon {\rm app}}}}}^{{2}}}$$

The coupled approach used in this study and its potential limitations was previously described in detail by Prochnow, et al.^29^ and is originally based on a concept published by Hepp, et al.^64^.

Our deuterium excess reconstruction indicates higher evaporative enrichment (= more negative deuterium excess) at the beginning of the Bølling-Allerød interstadial and a trend to reduced evaporation thereafter. The Younger Dryas and the Early Holocene are characterized by stronger evaporation, but pronounced short-term fluctuations in deuterium excess occur around ~ 11.5 and ~ 11.2 ka cal. BP during the Preboreal.

**Fig. 7:**
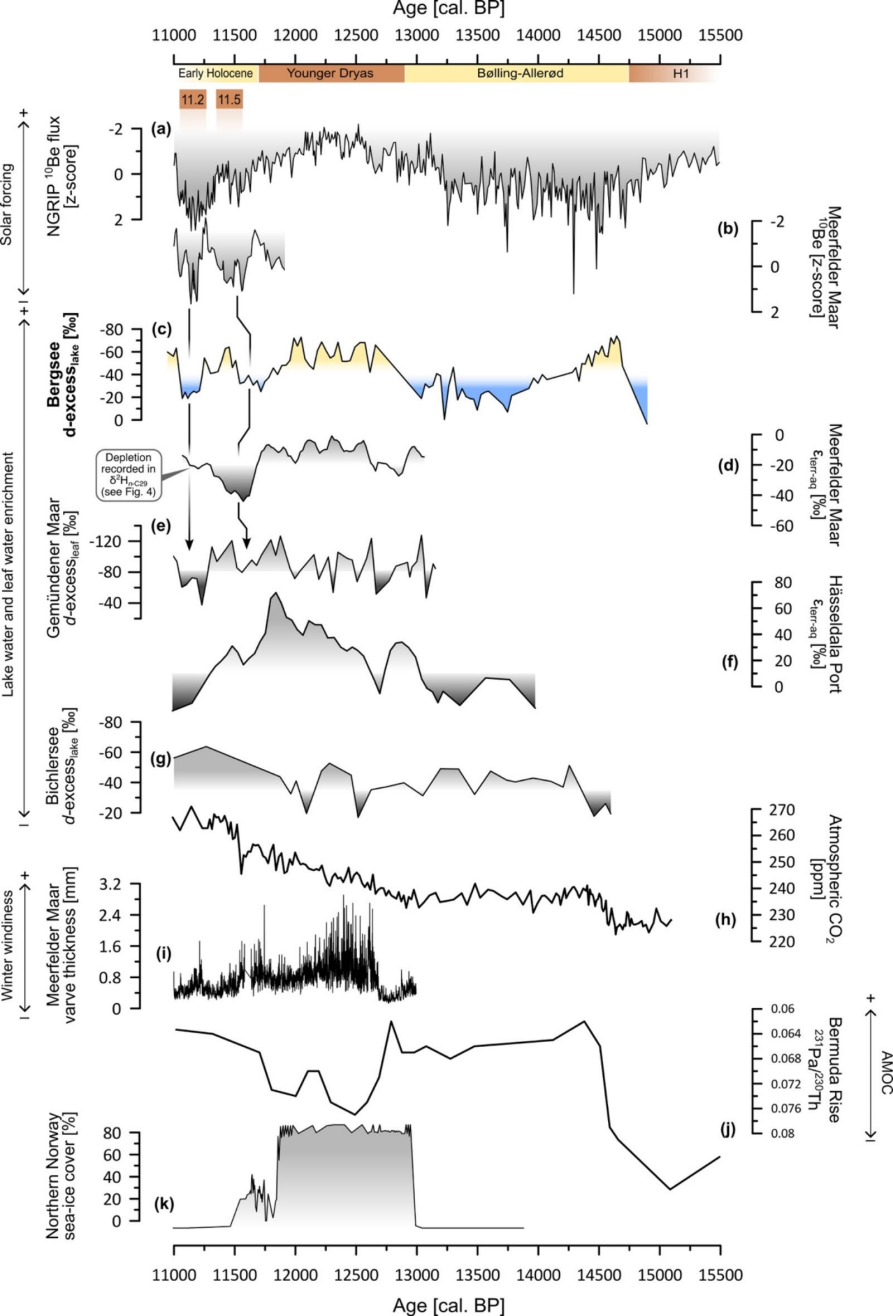
Compilation of paleohydrological reconstructions across Europe. (**a**) NGRIP ^10^Be^90^ and (**b**) Meerfelder Maar ^10^Be^88,91^ as proxies for solar forcing show coincidence with short-term hydrological fluctuations during the Preboreal visible in records from (**c**) Bergsee, (**d**) Meerfelder Maar^15^ and (**e**) Gemündener Maar^13^ and (**f**) Hässeldala Port^14^. Plot (**g**) shows Bichlersee deuterium excess^29^. Atmospheric CO_2_ forcing^76^ is shown in (**h**). Meerfelder Maar varve thickness^81^ is plotted in (**i**) and indicates strong windiness over Central Europe during the Younger Dryas winters. The (**j**) Bermuda Rise ^231^Pa/^230^Th^80^ and (**k**) Northern Norway sea ice cover^92^ reflect environmental conditions in the North Atlantic. The figure was created with Inkscape 1.3.2 (www.inkscape.org).

Increased evaporative enrichment between 14.7 and 14.5 ka cal. BP is in good agreement with lowest lake levels at Gerzensee, Switzerland^74^, and increasing temperatures due to abrupt increasing CO_2_ concentrations at the onset of the Bølling-Allerød^75,76^. Lower deuterium excess at Bergsee after 14.5 ka cal. BP is confirmed by increasing lake levels at Gerzensee and might be related to lower solar forcing recorded by higher ^10^Be fluxes in Greenland ice cores (Fig. 7a). ^10^Be is a cosmogenic nuclide that is produced by spallation of atoms (mainly oxygen and nitrogen) in the atmosphere due to incoming cosmic radiation. On short time scales, ^10^Be production is modulated by solar activity, where higher solar activity results in less cosmic radiation penetrating earth’s atmosphere and a lower ^10^Be production rate. Thus, higher ^10^Be concentrations reflect minima in solar forcing. Regarding this, one might be tempted to compare the long-term, multi-millennial trends visible in the ^10^Be flux with the long-term trend in evaporative enrichment at Bergsee. However, this might be misleading as the long-term, millennial ^10^Be fluctuations are explained by geomagnetic field variations of the Earth rather than solar modulation^77^.

An increase in evaporative enrichment at Bergsee is visible during the transition from the Bølling-Allerød into the Younger Dryas at ~ 12.8 ka BP. This finding is again confirmed by lake level low stands in Switzerland^8^. Moreover, it agrees with higher evapo(transpi)rative enrichment inferred from a dual biomarker approach (ε_terr−aq_) at Meerfelder Maar and, despite its high variability, partly by deuterium excess at Gemündener Maar^13,15,23^. Similar results were also inferred from an additional biomarker ε_terr−aq_ record at Hässeldala port (Fig. 7f) in south Sweden^14^. Overall, these biomarker isotope datasets point out that Younger Dryas summers were relatively dry (Fig. 7c-f). Drier conditions were independently reconstructed based on triple oxygen and hydrogen isotopes in the Pyrenees^78^, whereas lower annual paleoprecipitation during the Younger Dryas was inferred from Scandinavia to the Alps^79^.

The Younger Dryas cooling is usually attributed to an increase of meltwater input from the retreating ice sheets in North America and a successive slowdown of the Atlantic Meridional Overturning circulation (AMOC; Fig. 7j) in the North Atlantic^4,80^. The cooling pushed the Westerlies further south, resulting in drier and windier conditions particular during winter (Fig. 7i) in Central Europe^81^. However, there are doubts whether this meltwater mechanism is sufficient enough to explain seasonal differences in Central Europe, especially for summer^5^. Seasonal sea ice cover was much stronger (Fig. 7k), and SST dropped markedly during the Younger Dryas^5,31,82,83^. These specific conditions in the North Atlantic realm resulted in a more zonal flow of the Westerlies during the long and cold Younger Dryas winters. In summer, however, the strong SST gradient in the North Atlantic realm favored a high-pressure belt between the Azores and the polar ice sheet, causing strong atmospheric blocking over Central Europe during the short but warm Younger Dryas summers^5^. This mechanism probably dampened the cooling induced by the AMOC slowdown^5^. Rather than only a southward shift of the Westerlies during winter, we argue that this seasonal atmospheric difference is a plausible explanation for the overall dry conditions inferred by the summer-sensitive biomarker proxies across Central Europe during the Younger Dryas. This atmospheric pattern is very similar to multidecadal atmospheric summer oscillations in the North Atlantic^84^ and agrees with recent observations of strong heat waves in Central Europe in relation to unusually cool winter SST anomalies in the North Atlantic^85^. On a broader spatial scale, summer blocking over Central Europe caused positive precipitation anomalies and cold temperatures in southeastern Europe and the eastern Mediterranean realm^79^. This might also explain the strong southward extent of permafrost in the Ural Mountains during the Younger Dryas^86^.

Another deuterium excess record spanning the Late Glacial and Early Holocene was established at Bichlersee^29^, a small mountain lake in the Northern Alps, ~ 200 km east of Bergsee (Fig. 7f). Like at Bergsee, the Bichlersee record shows enhanced enrichment during the onset of the Bølling-Allerød but a particular difference during the Younger Dryas is not visible. One reason might be a potential bias due to winter precipitation as the Bichlersee catchment is characterized by karst and located at higher altitude favoring snow cover. However, the Bichlersee record has significant lower resolution as the other records, so a more detailed comparison is limited.

A last aspect to be discussed is the short-term variability of our Bergsee deuterium excess record. The deuterium excess suggest two periods of lower evaporative enrichment during 11.5 and 11.2 ka cal. BP. An “11.5 ka-event” is already known from Greenland ice cores as the so-called “Preboreal Oscillation” with a pronounced negative δ^2^H excursion indicating cooler conditions^4,87^. Lower evaporation at Bergsee is also independently supported by lake level highstands in the Western Alps^9^. Our data compilation in Fig. 7 suggest that this wetter period seems to be recorded in ε_terr−aq_ from Meerfelder Maar and Hässeldala Port as well as deuterium excess at Gemündener Maar, albeit the Preboreal Oscillation is absent in the raw leaf wax δ^2^H data from Bergsee, Meerfelder Maar and Gemündener Maar^88^. The Preboreal Oscillation was attributed to a slow-down of the AMOC due to a meltwater outburst from the remaining Laurentide Ice Sheet, leading to cooler conditions^89^. In terms of solar forcing, there seems to be a minor reduction in solar activity visible in ^10^Be from Meerfelder Maar, but not particular in ^10^Be from Greenland. However, at least according to our known literature, the exact cause of this climate oscillation is not resolved yet.

A second oscillation succeeding the Preboreal Oscillation only a few hundred years later was described in Meerfelder Maar and Gemündener Maar around ~ 11.2 ka cal. BP. It was called “Meerfelder Maar Oscillation” or “Preboreal Humid Phase” and indicates again cooler and wetter conditions^13,88^. A coincidence between ^10^Be and low evapo(transpi)rative enrichment can be seen during these Preboreal climate oscillations in all three biomarker records from Bergsee, Meerfelder Maar and Gemündener Maar, providing evidence that evapo(transpi)rative enrichment is to some degree controlled by solar forcing; at least during the Meerfelder Maar Oscillation/Preboreal Humid Phase. Although age uncertainties ( ~ ± 300 years) of the Bergsee record are relatively high after ~ 11.2 ka cal. BP, the coincidence of lower solar forcing with reduced evaporation is the most likely explanation for the pattern observed at Bergsee, as similar relationships were also observed at other sites^13,24^.

But how does seasonality during these short abrupt periods affect hydrology? Solar forcing is probably relatively constant throughout a year and fluctuates considerable only over a period of several years, thus it has no seasonal bias. Like for the Younger Dryas, cooling seems important during winter also during the Preboreal climate fluctuations. We speculate that a combination of a longer and cooler winter season together with dampened solar insolation caused lower evapo(transpi)rative enrichment during these periods.

## Conclusion

We coupled a high-resolution *n*-alkane δ^2^H and hemicellulose sugar δ^18^O dataset based on lake sediments from Bergsee, Southern Germany, in order to reconstruct deuterium excess as a proxy for evaporative enrichment during the Late Glacial and Early Holocene.

At Bergsee, the *n-*alkane *n*-C_31_ is mainly produced by grasses and its δ^2^H signal records the isotopic composition of precipitation. Hemicellulose sugars are of aquatic origin and their δ^18^O signal documents the isotopic composition of lake water, which is driven by evaporative enrichment.

In combination with our Bergsee data, a compilation of Central European biomarker stable-isotope records reveals a consistent pattern during the Late Glacial. However, they all lack a strong isotopic depletion during the Younger Dryas cool period. We suppose that this discrepancy is best explained by the seasonal sensitivity of the biomarker proxies. While these biomarker stable-isotope records primarily reflect a summer signal, and because Younger Dryas summers were relatively warm, there is an absence of the strong winter cooling signals, which are present in annual water isotope records like Greenland or Lake Steißlingen. However, a weak depletion during the Younger Dryas and a short-term variability is registered in some of the biomarker records, which might be explained by a winter bias depending on the specific hydrological setting of each lake. In summary, this emphasizes the importance of seasonality when comparing biomarker stable isotopes with other proxies.

We calculated deuterium excess as a proxy for evaporative enrichment at Bergsee by coupling δ^2^H and δ^18^O and to compare it with other European biomarker evapo(transpi)ration reconstructions. Results draw a consistent picture of paleohydrology in Central Europe during the Late Glacial, highlighting the robustness of the dual and coupled isotope approach to reconstruct evapo(transpi)ration. Under consideration of the summer bias, they provide strong evidence for dry hydroclimate conditions during the warm Younger Dryas summers. We suggest that a recently proposed feedback mechanism between North Atlantic sea ice, strong winter cooling and summer atmospheric blocking over Central Europe is a suitable explanation for this signal, while a previously proposed southward shift of the Westerlies, which is particular a winter signal, can probably not solely explain drier summers. The overall agreement even of short-term fluctuations in evapo(transpi)rative enrichment documented by biomarker stable isotope paleohydrology around ~ 11.5 and ~ 11.2 ka cal. BP adds additional confidence to the robustness of all these biomarker records and their coincidence with ^10^Be excursions found in Greenland ice cores suggest a partial control of solar forcing on paleohydrology.

Our study highlights the great advantages of multi-isotope approaches compared to single isotope studies in paleohydrology as both dual and coupled isotope approaches allow a more detailed differentiation between isotopic effects on precipitation and evapo(transpi)ration. Nevertheless, a careful evaluation of aquatic versus terrestrial biomarker sources is needed for a robust interpretation of such records.

## Electronic Supplementary Material

Below is the link to the electronic supplementary material.

Supplementary Material 1

## Data Availability

The data generated in this study is shared by a supplementary spreadsheet.
